# Health system–community partnerships in a Medicaid nutrition and housing program, 2020-2023: a mixed-methods evaluation

**DOI:** 10.1093/haschl/qxag179

**Published:** 2026-07-18

**Authors:** Jessica L McCurley, Vicki Fung, Douglas E Levy, Sydney McGovern, Christine Vogeli, Cheryl R Clark, Anne N Thorndike

**Affiliations:** Department of Psychology, San Diego State University, San Diego, CA 92182, United States; Harvard Medical School, Boston, MA 02115, United States; Mongan Institute Health Policy Research Center, Massachusetts General Hospital, Boston, MA 02114, United States; Harvard Medical School, Boston, MA 02115, United States; Mongan Institute Health Policy Research Center, Massachusetts General Hospital, Boston, MA 02114, United States; Division of General Internal Medicine, Massachusetts General Hospital, Boston, MA 02114, United States; Harvard Medical School, Boston, MA 02115, United States; Mongan Institute Health Policy Research Center, Massachusetts General Hospital, Boston, MA 02114, United States; Harvard Medical School, Boston, MA 02115, United States; Division of General Internal Medicine & Primary Care, Brigham and Women's Hospital, Boston, MA 02115, United States; Institute for Health Equity Research, Evaluation, and Policy, Massachusetts League of Community Health Centers, Boston, MA 02108, United States; Harvard Medical School, Boston, MA 02115, United States; Division of General Internal Medicine, Massachusetts General Hospital, Boston, MA 02114, United States

**Keywords:** Medicaid, health-related social needs, food insecurity, housing insecurity, accountable care organization, social service organization, medically tailored meals, produce vouchers, housing assistance, implementation evaluation, mixed methods

## Abstract

**Introduction:**

Health systems are partnering with social service organizations (SSOs) to establish health-related social needs (HRSN) programs, yet best practices have not been established.

**Methods:**

This study assessed the implementation of a partnership between one accountable care organization (ACO) and SSOs in Massachusetts' Medicaid Flexible Services Program (FSP) from 2020 to 2023 to address food and housing insecurity. To be FSP-eligible, individuals needed to be in the ACO, have food or housing insecurity, and meet specific health criteria. Quantitative measures of reach, enrollment, and services were integrated with findings from interviews with ACO (*n* = 12) and SSO (*n* = 11) staff.

**Results:**

Overall, 15.1% of 16 289 FSP-eligible adults and 11.8% of 5476 FSP-eligible children were enrolled. Nutrition enrollments increased over time, but housing enrollments remained low. ACO and SSO staff described positive impacts, including tailored support for patients' needs, SSO organizational growth, and understanding of how SSOs can partner with health care. Implementation challenges included complex contracts, administrative burden, and housing shortages. State funding for SSO infrastructure, cross-sector data sharing, workflow simplification, and ACO–SSO communication improved efficiency and reduced burden.

**Conclusion:**

The ACO–SSO partnerships expanded resources to address beneficiaries' HRSN. Understanding factors that contribute to success on both sides of the partnership can inform future HRSN programs.

## Introduction

Many US health care organizations have implemented health-related social needs (HRSN) programs to help prevent and treat chronic disease, reduce healthcare costs, and address health inequity. To accomplish these goals, some health systems establish formal partnerships with community-based social service organizations (SSOs) and financial contracts to pay SSOs for services rendered to their patients.^1^ SSOs serve as essential partners to health systems as they are known and trusted entities that have the operational capacity and expertise to deliver targeted social needs interventions in their communities. However, developing sustainable cross-sector partnerships between SSOs and health systems is complex and resource-intensive, with inherent challenges in data sharing, closed-loop communication, and implementation of intervention protocols.^2-5^ Identifying factors that facilitate these partnerships can guide health care organizations in the implementation, adoption, and maintenance of HRSN programs over time.

Health system-based HRSN interventions have the potential to improve food and housing security, but the design and duration of these programs vary greatly, their impact on health outcomes is variable, and implementation is understudied.^3,6-11^ Increasingly, policy approaches to addressing HRSN are being implemented as part of payer and health system strategies to address poor health outcomes and disparities driven by socioeconomic inequality. As of January 2026, Medicaid agencies in 25 states have received approval for Section 1115 demonstration waivers to use Medicaid funding to address HRSNs such as food insecurity and housing instability.^12^ In March 2025, the Centers for Medicare & Medicaid Services rescinded prior guidance encouraging the submission of new Section 1115 demonstration waivers addressing HRSN; however, existing waivers have remained in place.^13^ Many of these programs include formal partnerships between health systems and SSOs, often with financial contracts to pay SSOs for services delivered.

In 2017, Massachusetts Medicaid (MassHealth) initiated accountable care organizations (ACOs), and in 2020, MassHealth launched a pilot of the Flexible Services Program (FSP), a $149 million initiative that provided funding to ACOs statewide to contract with SSOs to address food and housing insecurity among eligible Medicaid patients. FSP was authorized through a Section 1115 demonstration waiver and funded through a Medicaid performance-based Delivery System Reform Incentive Payment.^14^ A previous study explored the first 17 months of FSP implementation from the perspectives of one ACO's patients and health system staff.^2^ The current study examined FSP implementation over the full 3-year pilot (March 2020 to March 2023), focusing on the benefits and complexities of cross-sector contracts and incorporating firsthand perspectives from staff within participating SSOs and the ACO. Using mixed methods, findings from quantitative FSP enrollment data were integrated with qualitative interviews with ACO and SSO stakeholders, focusing on their experiences with implementing FSP. The objectives were to identify key challenges and solutions for successfully implementing partnerships between the ACO and SSOs.

## Methods

This study was conducted in the Mass General Brigham (MGB) Medicaid ACO and partner SSOs in Massachusetts from March 2020 to March 2023. A mixed-methods evaluation of FSP implementation was a planned component of *LiveWell/ViveBien*, a prospective cohort study guided by the RE-AIM/PRISM framework^15^ to assess outcomes associated with FSP participation in a sample of MGB health center patients.^2,16^ While a prior study addressed reach, adoption, implementation, and contextual factors during the first year and a half of the program,^2^ the current study used a convergent parallel design to evaluate the implementation and reach of FSP from the perspective of the ACO and SSOs engaged in financial contracts to deliver nutrition and housing services to eligible Medicaid beneficiaries. The roll-out of FSP in this ACO began in March of 2020; in anticipation of this start date, the ACO began implementing systematic screening for food and housing insecurity in March 2018.^2,17^ Quantitative data presented are for the initial 3-year pilot period (March 2020 to March 2023). Qualitative interviews were conducted in 2023 and discussed the implementation of FSP from March 2020 to March 2023. The MGB institutional review board approved all study procedures on August 27, 2019. The study and manuscript adhere to the Standards for Reporting Qualitative Research guidelines.^18^

### Flexible Services Program

FSP consisted of 2 categories of services: (1) nutrition sustaining supports (eg, provision of healthy food boxes, produce vouchers, medically-tailored meals, or nutrition education), and (2) tenancy preservation supports, which included pre-tenancy support (eg, assistance with affordable housing applications), tenancy sustaining support, transitional assistance, and home modifications. To be eligible for FSP, ACO-enrolled patients must have had food or housing insecurity and met at least one health needs-based criterion (eg, complex physical health need such as uncontrolled diabetes, or behavioral health need such as depression). Health system staff enrolled patients by identifying individuals with food and housing insecurity as assessed by annual screening or provider referral, confirming inclusion criteria via electronic medical record, and completing referral paperwork. Each ACO was allotted an annual budget for FSP based on the number of Medicaid beneficiaries enrolled and in accordance with the ACO's approved budget and FSP participation plan.^14^ FSP was not an entitlement or mandatory benefit; it was designed as a pilot program to serve some, but not all, eligible patients. Each ACO had the discretion to develop protocols for identification and enrollment of members, allowing them to target their ACO members who might benefit most. Participating ACOs were allowed to select and contract with SSOs who were best suited to provide nutrition and/or housing services to their patient population, based on the SSO's geographic location, expertise, and capacity for service provision. Technical assistance for FSP participation was provided to some SSOs through grant funding offered by MassHealth.^14^

### Quantitative data and analyses

Reach was evaluated with descriptive statistics of the number, proportion, and demographics of Medicaid ACO adults and children enrolled in FSP compared to those who were eligible but not enrolled, as documented in the health system's electronic medical record and FSP administrative records. For this analysis, patients were considered eligible if they were aligned to the Mass General Brigham ACO, screened positive for food or housing insecurity, and had at least one complex health condition that met criteria for FSP. The number of ACO–SSO partnerships established, the proportion of enrollments by service type (nutrition or housing), the mean duration of FSP enrollment, and the number of new enrollments in each service type per quarter were also assessed.

### Qualitative data and analyses

Implementation of FSP contracts was assessed through qualitative interviews with ACO and SSO staff involved in program operations. Staff were recruited for interviews through purposive sampling based on their role in directing or implementing FSP. A semi-structured interview guide (Supplement A) prompted discussion of challenges and solutions as well as the organizational impact of FSP. Interviews were conducted by phone or video call and were audio-recorded and transcribed. Thematic qualitative analysis was conducted following the Framework Method^19^ using Dedoose software, version 9.0 (SocioCultural Research Consultants, LLC). Both deductive coding (using a priori codes based on prior research) and inductive coding (allowing for the emergence of new codes) were utilized to create the coding protocol. Two trained research assistants coded all transcripts independently and compared codes. A Ph.D.-level researcher (J.L.M.) provided oversight and resolved coding discrepancies. Results were reviewed with the interdisciplinary research team (primary care physicians, health services researchers, clinical psychologist) and select interviewees to ensure face validity. Verbal consent for participation and use of interview content was provided by all participants.

### Data integration

When quantitative and qualitative analyses were complete, findings were integrated by developing a joint display^20^ with quantitative data on FSP reach and implementation, qualitative data summarizing implementation challenges and solutions, and integrated insights and recommendations. The integrated insights^20^ emerged through the convergence of quantitative and qualitative data strands and offer a more comprehensive interpretation of results.

## Results

### Quantitative results: reach and implementation

**Figure 1.**
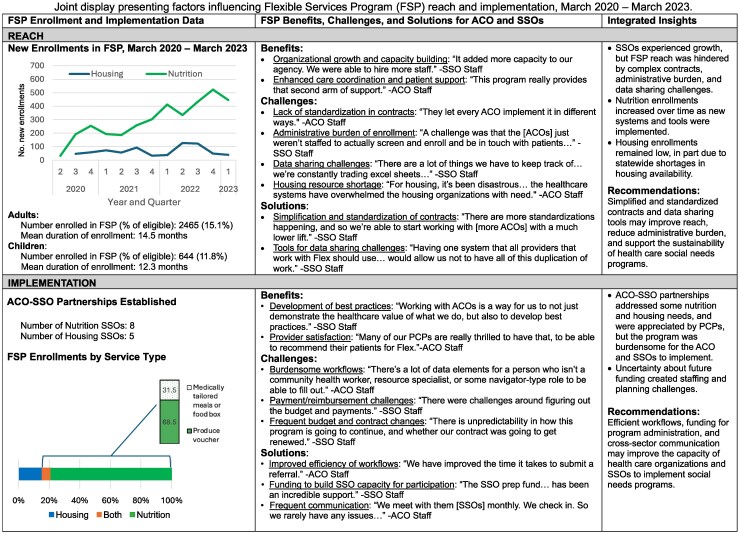
Joint display (see separate file).

Of 16 289 adult ACO members who were eligible for FSP, 2465 (15.1%) were enrolled (Table 1), and mean duration of FSP enrollment was 14.5 months (Figure 1). Among 5476 eligible children, 644 (11.8%) were enrolled (Table 1), and mean duration was 12.3 months (Figure 1). Comparing FSP-enrolled to non-enrolled FSP-eligible adults, there was a higher proportion that were female (74.8% vs 67.4%), Hispanic (43.4% vs 34.5%), and Black (21.2% vs 16.5%). Among FSP-enrolled children, there was a higher proportion that were Black (16.1% vs 11.9%) and Hispanic/Latino (56.6% vs 53.2%) than among non-enrolled children (Table 1). Overall, 51.3% of all ACO members completed at least one screener for food or housing insecurity between March 2018 and March 2023; among those enrolled in FSP, 93.7% of adults and 96.0% of children completed at least one screener (Table S1). The most common FSP-eligible medical conditions of adults enrolled in FSP were obesity, depression, anxiety, hypertension, and diabetes; the most common conditions for children enrolled in FSP were obesity, neurodevelopmental disorders, failure to thrive/nutritional disorders, asthma, and anxiety (Table S2).

**Table 1.** Demographic characteristics of potentially eligible^a^ Medicaid Accountable Care Organization enrollees and Flexible Services enrollees aligned to the Mass General Brigham ACO, 2020-2023.

	Medicaid ACO enrollees eligible for FSP^a^				
	Total eligible^a^	Eligible^a^ adults (≥ 21 years)	Adults enrolled in FSP	Eligible^a^ Children (<21 years)	Children enrolled in FSP
N	21765	16289	2465	5476	644
Age, mean [SD]	39.97 [18.3]	43.35 [12.5]	44.57 [12.0]	10.07 [6.3]	9.55 [6.2]
Sex, N (%)					
Female	13691 (62.9)	10980 (67.4)	1869 (75.8)	2711 (49.5)	335 (52.0)
Male	8074 (37.1)	5309 (32.6)	596 (24.2)	2765 (50.5)	309 (48.0)
Ethnicity, N (%)					
Hispanic	8536 (39.2)	5618 (34.5)	1118 (45.4)	3918 (53.3)	365 (56.7)
Non-Hispanic	11969 (55.0)	10031 (61.6)	1284 (52.1)	1938 (35.4)	216 (33.5)
Unknown	1260 (5.8)	640 (3.9)	63 (2.6)	620 (11.3)	63 (9.8)
Race,^b^ N (%)					
American Indian or Alaska Native	72 (0.3)	59 (0.4)	—	—	—
Asian	596 (2.7)	468 (2.9)	36 (1.5)	128 (2.3)	12 (1.9)
Black	3332 (15.3)	2689 (16.5)	522 (21.2)	643 (11.7)	104 (16.2)
Native Hawaiian or Pacific Islander	24 (0.1)	17 (0.1)	—	—	—
White	9450 (43.4)	7902 (48.5)	882 (35.8)	1548 (28.3)	156 (24.2)
Other^c^	5600 (25.7)	3641 (22.3)	734 (29.8)	1959 (35.8)	249 (38.7)
Mixed	712 (3.3)	488 (3.0)	80 (3.3)	224 (4.1)	33 (5.1)
Unknown	1979 (9.1)	1025 (6.3)	201 (8.2)	954 (17.4)	89 (13.8)

Table Notes: ACO=Accountable Care Organization; Flex=Flexible Services program; — denotes <14 individuals.

^a^Eligible patients were those who were aligned to the Mass General Brigham ACO, screened positive for food and/or housing insecurity at some point during the Flex pilot, and had a complex health condition (e.g., uncontrolled diabetes, depression) identified in the electronic medical record that satisfied eligibility criteria for Flex eligibility.

^b^Race was self-reported in the electronic health record (EHR) data. All categories from the EHR are included in the table.

^c^82% enrollees in this category reported Hispanic ethnicity.

The ACO established formal partnerships and financial contracts with 13 SSOs (8 nutrition service providers, 5 housing service providers). The joint display (Figure 1) shows the number of new FSP enrollments in nutrition and housing services per quarter during the pilot period (March 2020 to March 2023). Most enrollments were for nutrition services (*n* = 3565, 83.0%), which included medically tailored meals or food boxes (*n* = 1141, 26.6%) and produce vouchers (*n* = 2424, 56.4%). Some patients were enrolled in both nutrition and housing services (6.3% of adults, 4.2% of children); and the mean number of FSP enrollments was 1.4 (SD: 0.7) per adult and 1.3 (SD: 0.6) per child (Figure 1, Table S3).

### Qualitative results

Benefits, challenges, and solutions that impacted FSP reach and implementation, as reported by ACO and SSO staff, are presented in Figure 1. SSO staff described many positive impacts, including organizational growth, service expansion, capacity building, development of best practices, and enhanced understanding of the health care value of their services and how to partner with health care organizations. SSO staff reported that participation in FSP enabled their organizations to expand capacity by hiring additional personnel, serving a larger number of individuals with nutrition and housing needs, and, in some cases, broadening the scope of services offered. Many described participation as both a privilege and a valuable opportunity to collaborate with health care partners toward a shared goal of improving patient well-being. For some organizations, successful implementation of one ACO partnership facilitated expansion to additional ACO collaborations, further increasing revenue and service reach. Some SSOs described receiving grants awarded by MassHealth to support building their organizational infrastructure for FSP administration. Those who received these funds used them for critical preparation tasks, such as hiring new staff, adjusting service offerings and delivery models for FSP, establishing operational procedures, building HIPAA-compliant tracking and data sharing systems, and participating in state-wide planning meetings.

ACO staff also described multiple benefits of FSP. They shared that physicians, particularly primary care providers, valued having FSP resources available for their patients. FSP program management staff appreciated having the flexibility to contract with SSOs that offered the best-fitting services for their patients. Staff in other care management team positions (eg, social workers, care navigators) expressed enthusiasm for FSP as an additional layer of support for patients and reported that increased communication with SSO staff improved care coordination after patients were enrolled in the program.

Challenges that impacted FSP reach included complex contracts, administrative burden, data sharing challenges, and statewide housing resource shortages. MassHealth established general guidelines for FSP implementation but allowed all ACOs to individualize contract terms and parameters with their selected SSOs. While this provided ACOs’ flexibility to select their preferred SSOs and establish contract terms, it led to wide variation in contracting practices. Since many SSOs managed contracts with multiple ACOs, SSOs explained that managing each contract's service and reimbursement terms added time-consuming paperwork and adjustment of services provided. Both ACO and SSO staff noted that enrollment processes were time-consuming and at times overwhelming, contributing to overload and inability to enroll more patients.

Further impacting the program's reach, both SSOs and the ACO experienced challenges with data sharing. Because the ACO was responsible for tracking FSP enrollees' outcomes, closed feedback loops were needed but were difficult to implement. For example, SSO staff described having to utilize multiple tracking databases or systems (eg, referral system mandated by each ACO as well as the SSO's own internal tracking database), leading to redundant and inefficient documentation processes. Because ACO-mandated tracking systems were burdensome, SSO staff did not always complete them according to protocol, leading to the inability to track enrollees' status or outcomes. Finally, both the ACO and SSOs reported that because of a statewide shortage of affordable housing options and long wait times for subsidized housing, housing SSOs were overwhelmed with FSP enrollees for whom there were few immediate service options and could not serve additional enrollees.

SSOs and the ACO also noted ongoing challenges to implementation (Figure 1). Both SSOs and the ACO reported burdensome workflows and a lack of staff bandwidth. For ACO staff, a time-consuming administrative task was quarterly and annual reporting requirements for MassHealth, which included details about eligibility, enrollment, and receipt of services for every FSP enrollee, as well as a detailed count of program expenditures. SSO staff recounted challenges related to budgeting and payment for their services, including delayed reimbursements and payment models that were a poor fit for their operations. For example, while invoice-to-payment cycles of 30 days or more were common for the health system, SSOs had difficulty supporting operations through long reimbursement delays. For SSOs whose services included farm-sourced produce boxes, for example, retrospective fee-for-service payment models were suboptimal, as they had to forecast produce needs many months ahead. On the other hand, prospective payment models lacked flexibility to accommodate fluctuations in referral volume and thus constrained SSOs' ability to be responsive to shifts in demand for services. Further, FSP contracts were subject to annual changes in terms and budgets based on programmatic and policy shifts enacted by MassHealth and the ACO. SSO staff identified the year-to-year uncertainty of contracts as a major operational challenge that led to difficulty ensuring the sustainability of the program, including staffing and resource management.

SSOs and the ACO described important solutions implemented to address some of these challenges. SSO staff noted that simplified contracts reduced operational burden and facilitated adoption of new contracts with additional ACOs. SSOs recommended careful consideration of payment models and reimbursement schedules to fit their operational needs and avoid adverse economic outcomes or reduced service capacity. Tools to address data sharing challenges, such as crosswalks between the ACOs’ and SSOs' data tracking systems, reduced duplication of work but were expensive and time-consuming for some to develop. ACO staff reported that increasing standardization and efficiency of workflows (eg, simplifying enrollment processes) reduced staff time burden to implement FSP, but also took time to develop. SSOs who benefited from preparation grant funds awarded by MassHealth to support organizational infrastructure for FSP administration recommended that these preparation funds be universally available.

Routine communication (eg, monthly ACO–SSO meetings) was cited as a key to improving program operations. Both ACO and SSO staff emphasized the importance of regular cross-sector communication, including more direct communication from the ACO and MassHealth about any programmatic and budget changes. Several ACO staff recommended more support and unified strategies from the state to address common implementation challenges.

### Integrated insights

Converging quantitative and qualitative data on the enrollment, reach, and implementation of FSP led to important insights and recommendations for future health system–SSO linkages. While FSP had multi-level benefits, such as promoting growth and organizational capacity for SSOs, providing an important resource for primary care providers, and addressing food and housing needs of some patients, the program's reach was limited by administrative complexity, operational burden, and data sharing challenges. Annual budget changes and uncertainty about the program's future made it difficult for both SSOs and the ACO to plan and staff the program appropriately. Nutrition enrollments increased steadily during the pilot's 3-year timeline as systems and workflows became established, while housing enrollments remained low, in part due to a state-wide shortage of affordable housing units. Several initiatives helped reduce the program's burden and increase its reach, including efforts to simplify and standardize contracts and workflows, development and implementation of tools for optimal data sharing, state funding to support SSO capacity for participation, and maintaining consistent ACO–SSO communication.

## Discussion

This study in one ACO in eastern Massachusetts demonstrated that approximately 1 in 7 potentially eligible Medicaid ACO participants were enrolled in FSP over 3 years, and mean duration of enrollment was just over 1 year. Although overall enrollment represented a relatively small proportion of the eligible population, nutrition services increased steadily throughout the program. FSP was not designed as an entitlement program that could enroll all eligible individuals; however, factors related to the SSO–ACO partnerships may have limited the ability of the program to enroll more participants, particularly in initial months. Qualitative interviews with SSO and ACO staff identified challenges and successes that likely impacted the program's reach, implementation, and impact of FSP on the participating organizations. While the typical FSP enrollment period was 6 months, participants could be re-enrolled for an additional 6 months or more if they remained eligible. Staff attributed longer enrollments to patients' persistent food or housing needs and availability of unspent program funds in the program's first 2 years. As the pilot progressed, simplification and standardization of ACO–SSO contracts, tools to address data sharing challenges, and workflow efficiencies improved implementation, but the operational burden of the program—even at a relatively low rate of enrollment—could impede scaling and sustainability in the future.

The challenges and solutions experienced by this ACO and its SSO partners may be shared by other ACOs and health care systems nationally. Recent publications evaluating this and other health system-based multisector collaborations to address social needs cited similar challenges, such as high costs, staff shortages to implement programs, variable capacity of community partners, and similar solutions or best practice recommendations, such as financial support for community partners, bidirectional communication, and infrastructure for effective program administration.^3-5,21^

Participating in FSP had mixed impact on the SSOs and the ACO, demonstrating that health system-community partnerships and contracts for social care provision can have organizational benefits as well as substantial hidden costs. As reported by SSOs in this study, partnering with health care organizations can lead to powerful organizational growth and development for community organizations.^3^ Developing scalable delivery models, understanding the health care system, and recognizing the health impact of their services can be transformative for small organizations and the individuals they serve. Inside health care systems, direct care providers may be enthusiastic about having HRSN referral options for their patients. In this study, the SSOs reporting benefits were often recipients of state grant funding to prepare for FSP. Approximately $4 million was allocated by MassHealth to support SSOs preparing for FSP, but only SSOs with existing ACO relationships in 2020 were eligible; SSOs that contracted with ACOs later in the pilot timeline were ineligible.^22^ While structured funding opportunities may enhance benefits and mitigate challenges for SSOs, they introduce additional administrative costs that policy makers must consider.

The impact of HRSN programs on patients' short- and long-term health outcomes has been variable, with many programs reporting little to no impact on social needs or health,^3,7^ or lack of maintenance of health benefits once the intervention is discontinued,^23^ while the financial and operational investments are steep. Results of other HRSN interventions have suggested that certain services—produce subsidies, for example—were more impactful on health outcomes than others (eg, food boxes).^24^ A small cohort study that assessed changes in food and housing insecurity, dietary intake, stress, and acute health care utilization among individuals from one ACO enrolled in FSP found no improvements in these outcomes.^16^ Another study that examined health care use and costs statewide during the 3-year FSP pilot found a small reduction in hospitalizations and emergency department visits for those who were enrolled in FSP, but no significant changes in health care costs.^25^ It remains unclear the extent to which implementation challenges and burden may have limited FSP's impact on health outcomes, and whether improved implementation of health system–SSO partnerships would result in improvements in HRSN and health outcomes.

In recent years, best practice recommendations for screening and addressing HRSN in health care settings have emerged and include critical components such as stakeholder engagement in program design, dedicated staffing, structured staff training, closed-loop referrals, data sharing infrastructure, and payment models that support both screening and HRSN service provision.^26-28^ These recommendations principally target health care systems, with little guidance on best practices for SSOs. Other studies have documented the potential for digital health technologies, such as electronic health record apps and tools, to standardize and streamline data collection and care coordination.^27,29,30^ However, implementation of best practices and new tools requires substantial upfront investment and workflow redesign and is resource-intensive to maintain. Health systems must identify sustainable funding streams, carefully examine feasibility and workflow solutions, and collaborate with stakeholders to ascertain which clinical populations will benefit most from HRSN services being delivered through the health care system.

While evaluating patients' nonmedical needs is an important component of a health care system's approach to improving health equity among vulnerable populations, intervening directly to address social needs may be too labor- and resource-intensive for some systems and represents only one of many important and impactful strategies.^31-35^ Given the substantial investment of institutional time and resources needed to launch multi-sector programs like FSP, careful evaluation of a program's anticipated benefit-cost ratio, including start-up costs and funding to increase capacity for administration, will be needed in the future to ensure a program's viability and sustainability.

Although the current study focused on the initial FSP pilot period, the program continued under MassHealth's 2022-2027 Section 1115 demonstration, with the goal of further integrating nutrition and housing services into the ACO-based managed care delivery systems. In 2025, the program was re-organized as part of MassHealth's managed care delivery system,^36^ and therefore the extent to which the program is maintained, and factors contributing to its long-term sustainability, are important areas for future investigation.

### Strengths and limitations

A strength of this study is the mixed methods design, which allows for integration of quantitative reach and implementation metrics with in-depth qualitative data, including SSO perspectives on the experience of implementing FSP. An important limitation of the study is the focus on a single ACO in an urban health system and its SSO partners. Contextual factors of both the health system and the SSOs (eg, urban vs rural setting, resources available) may have impacted the specific challenges faced or solutions derived. Therefore, there is likely to be heterogeneity in how FSP was implemented across the state. Findings from this study may not generalize to all ACOs and SSOs and should be considered together with findings from other health system evaluations and other ACOs. Partnerships that integrate city- or county-wide stakeholders and resources, such as government entities and financial support for affordable housing, may be promising alternative models.^37^ The findings may have been impacted by the COVID-19 pandemic, which resulted in a complex array of unique challenges, particularly in the program's first 2 years. Finally, as the results of this study pertain to the first 3-year FSP pilot, whether the facilitators and barriers identified will contribute to long-term maintenance or adoption by new ACOs and SSOs is unknown.

## Conclusion

ACO–SSO partnerships developed in FSP provided resources to address housing and nutrition needs for Medicaid beneficiaries with complex health needs, with about 1 in 7 eligible ACO beneficiaries enrolled. FSP was complex and administratively demanding for both the ACO and SSOs. Both ACO and SSO staff expressed strong enthusiasm for the program and described meaningful organizational benefits associated with participation. At the same time, implementation was complex and frequently accompanied by substantial administrative burden, which at times overwhelmed staff capacity. This study highlighted that establishing streamlined contracts and sustainable reimbursement models for service provision were key components to reduce complexity and administrative burdens for the SSOs. Before health care systems invest in the development of community partnerships to address HRSN, it may be useful to understand factors on both sides of the partnership that contribute to success, including an in-depth understanding of the overall costs. Findings of this research could help inform strategies to improve efficiency and sustainability of cross-sector partnerships for social needs.

## Supplementary Material

qxag179_Supplementary_Data
