# Supplementary material for: Health system–community partnerships in a Medicaid nutrition and housing program, 2020-2023: a mixed-methods evaluation
Source: Health Aff Sch. 2026 Jul 18;4(8):qxag179. doi: 10.1093/haschl/qxag179 (PMC13439125; doi:10.1093/haschl/qxag179)
Supplement: Supplementary file 1 [file qxag179_supplementary_data.zip › Supplement 1_Interview Scripts_3.31.26.docx]

**SEMI-STRUCTURED INTERVIEW SCRIPT: SSO STAFF**

**Date: _________________**

**Role of Interviewee / Warm-up**

1. What is the title of your job or position at _______ (SSO Name)?
2. How long have you been working in this job?
3. Can you tell me a little bit about the services that your organization provides, and what your specific job/role involves?
4. What is your role with regards to the MassHealth Flexible Services program? When did you start to get involved with the Flex program?

**Partnering with MGB for Flex Services**

5. What was it like for your organization to set up a partnership with MGB for Flexible Services?
   a) Barriers/problems with setup: what was challenging?
   b) What worked well in the initial start-up?

6. How was this partnership with the hospital organized? What was in your contract? E.g., how many patients did you agree to serve, how were payment structured, etc
7. What was the role of MassHealth is this process?
   a) Did you have contact with them? Instructions/directions from them? What was this like?

**Barrier and Facilitators**

8. Once the Flex Services program began, how did it go for your organization?
   a) What have been the primary barriers, challenges, or problems? Can discuss each of these areas:
      i. partnering with hospital
      ii. working with patients
      iii. financial contract/payments
      iv. MassHealth/state (if any contact mentioned above)
      v. communication/data sharing with hospital
      vi. tracking and documentation
      vii. other impact on your staff or organization
   b) What has gone well about this program?
      i. Any ways it benefitted your organization or services offered?
      ii. Impact on number of patients served?
      iii. Impact on the community at large?
   c) How does your organization communicate with the hospital about patients served (e.g, by excel, email, databases, tools?) and how is this going?
      i. Challenges, changes that made it easier?
   d) What has the impact of the Flex program been on your organization’s staffing?
   e) What are your organization’s experiences with the financial contract and payment?
   f) Were there services you offered through Flex that were not a good fit for patients, or had low attendance or uptake? Reasons why?

**Changes and Modifications**

9. Have there been any changes or adaptations to the program, either by your organization or by the health system, that were important? Or helped it to go better?
   a) What was the adaptation, why, by whom – what changed as a result?
10. Are there aspects Flex that were planned but are not happening?
11. What are your thoughts about the impact this is having on the people enrolled in Flex Services? What is the impact you have seen for them?
    a) What is the ideal length of time people should receive these services, given that unlimited services likely isn’t feasible? An amount of time to them to have a meaningful benefit?

**Similar Programs**

12. Have you participated in Flex or other programs/partnerships with other hospitals or health systems/ACOs? If so, how does the Flex program/working with this hospital compare?
    a) Consider: type of contract/payment, burden of logistical challenges, notable differences in barriers/facilitators

**SEMI-STRUCTURED INTERVIEW SCRIPT: ACO STAFF**

**Date: _________________**

**Role of Interviewee**

1. What is the title of your job or position?
2. How long have you been working in this job?
3. What is your role with regards to the Flexible Services (FLEX) program? When did you start working with FLEX?

**Intro questions (based on interviewee’s history with FLEX):**

4. Can you start by telling me how FLEX is going, recently? What have been some of the challenges, successes, or changes in the last year?

**FLEX Program Objectives (Recent History)**

5. What were the primary objectives for the FLEX program during the PAST YEAR?
6. Were these objectives met? Why or why not?

**Recent Challenges**

7. What have been the biggest challenges or difficulties with Flex recently? Probe challenges at **different levels:**
- State/MassHealth (administrative burden/regulatory requirements, budget changes, etc)
- ACO / leadership level
- RSO level
- SSOs (contracting, availability, services offered, cost)
- Patients

Ask about known challenges/barriers:

- Identifying eligible patients
- Staffing challenges
- Communication and data sharing (Find Help, documentation burden, etc)
- Enrollment caps/goals - challenges with meeting or exceeding them
- SSO-related challenges
- Data tracking / reporting

**Differences by hospital (ask when appropriate for staff position)**

8. Are you aware of differences in how FLEX is being implemented by hospital, or differences in the challenges we’ve talked about by hospital?

b) Reasons for these differences?

9. Are you aware of any major differences in how FLEX being implemented at MGB versus other ACOs?
   a. Reasons for these differences?

**Changes and Modifications**

10. What are the most important factors in FLEX running well, currently?
11. Are there aspects of FLEX currently that are not functioning as intended or as planned?
12. Have there been recent changes or modifications to FLEX, either by the state/MassHealth or here locally, that have been important?

**Similar Programs – if time allows**

13. How does implementing FLEX compare to other similar programs within the ACO? (If interviewee has this experience)
    - Consider: logistical challenges, barriers/facilitators
    - How does FLEX interact/coordinate with other similar programs that enroll similar patients and address similar needs?
