# Supplementary material for: Health system–community partnerships in a Medicaid nutrition and housing program, 2020-2023: a mixed-methods evaluation
Source: Health Aff Sch. 2026 Jul 18;4(8):qxag179. doi: 10.1093/haschl/qxag179 (PMC13439125; doi:10.1093/haschl/qxag179)
Supplement: Supplementary file 1 [file qxag179_supplementary_data.zip › Supplement 2_Supplemental Tables_7.16.26.docx]

**Supplemental Table 1.** Completion of annual screening for food and housing insecurity by adult and pediatric Medicaid Accountable Care Organization enrollees aligned to the Mass General Brigham ACO from March 2020 – March 2023.

|  | **Medicaid ACO enrollees^2^** | | | | |
| --- | --- | --- | --- | --- | --- |
|  | **Total** | **Adults**  **(≥ 21 years)** | **Adults enrolled in FSP^3^** | **Children**  **(<21 years)** | **Children enrolled in FSP^3^** |
| N | 146883 | 96254 | 2465 | 50629 | 644 |
| At least one SDOH screener complete^1^ (N, %) | 75280 (51.25) | 45503 (47.27) | 2310 (93.71) | 29777 (58.81) | 618 (95.96) |
| Ever screened positive for food or housing insecurity (N, %) | 27589 (18.78) | 18152 (18.86) | 2171 (88.07) | 9437 (18.64) | 541 (84.01) |
| Ever screened positive for food insecurity (N, %) | 21425 (14.59) | 14406 (14.97) | 2042 (82.84) | 7019 (13.86) | 510 (79.19) |
| Ever screened positive for housing insecurity (N, %) | 14765 (10.05) | 9956 (10.34) | 1196 (48.52) | 4809 (9.50) | 233 (36.18) |

Table notes: ACO=Accountable Care Organization; FSP=Flexible Services program

^1^Screening was completed during primary care health visits or through contact with patients’ primary care clinicians from March 2018 (beginning of annual social needs screening for Medicaid ACO patients) through March 2023. These results reflect screening that was documented in a designated social needs screening module of the electronic health record (EHR). Assessment of social needs may have also occurred via informal conversations between patients and clinicians or other health center staff and documented in the EHR in non-systematic ways; those assessments are not reflected here.

^2^Medicaid ACO enrollees were patients aligned to the Mass General Brigham (MGB) ACO.

^3^Eligibility criteria for FSP included: (1) alignment to the MGB Medicaid ACO, (2) food or housing insecurity identified by screening or clinical encounter, and (3) a complex health condition (e.g., uncontrolled diabetes, depression) identified in the electronic medical record that satisfied eligibility criteria for Flex eligibility.

**Supplemental Table 2.** Medicaid Accountable Care Organization enrollees^1^ who screened positive for food and/or housing insecurity^2^ during March 2018 – March 2023 and medical conditions indicating eligibility for Flexible Services (FSP).

| **FSP-eligible condition,**  **N (%)** | **Total**  **(N=27589)** | **Adults**  **(≥ 21 years) not enrolled in FSP^3^**  **(N=15981)** | **Adults**  **enrolled in FSP^3^**  **(N=2171)** | **Children**  **(<21 years) not enrolled in FSP^3^**  **(N=8896)** | **Children enrolled in FSP^3^**  **(N=541)** |
| --- | --- | --- | --- | --- | --- |
| Any eligible condition | 21765 (78.89) | 14174 (88.69) | 2115 (97.42) | 5016 (56.38) | 460 (85.03) |
| Obesity | 13186 (47.79) | 9237 (57.80) | 1672 (77.02) | 1991 (22.38) | 286 (52.87) |
| Hypertension | 5734 (20.78) | 4718 (29.52) | 940 (43.30) | 62 (0.70) | 14 (2.59) |
| Diabetes | 3073 (11.14) | 2442 (15.28) | 596 (26.21) | 49 (0.55) | 13 (2.40) |
| Depression | 7565 (27.42) | 5579 (34.91) | 1122 (51.68) | 790 (8.88) | 74 (13.68) |
| Anxiety | 7950 (28.82) | 5872 (36.74) | 979 (45.09) | 1009 (11.34) | 90 (16.64) |
| Substance use disorder | 2282 (8.27) | 1941 (12.15) | 299 (13.77) | 40 (0.45) | --- |
| High-risk pregnancy | 397 (1.44) | 288 (1.80) | 97 (4.47) | --- | --- |
| Asthma | 4606 (16.70) | 2661 (16.65) | 471 (21.70) | 1357 (15.25) | 117 (21.63) |
| Neurodevelopmental disorder | 4063 (14.73) | 1501 (9.39) | 172 (7.92) | 2185 (24.56) | 205 (37.89) |
| Congestive heart failure | 690 (2.50) | 546 (3.42) | 131 (6.03) | --- | --- |
| COPD | 681 (2.47) | 569 (3.56) | 111 (5.11) | --- | --- |
| Chronic kidney disease, Stage 4 or 5 | 767 (2.78) | 584 (3.65) | 169 (7.78) | --- | --- |
| Failure to thrive/ nutritional deficiencies | 5078 (18.41) | 3477 (21.76) | 747 (34.41) | 747 (8.40) | 107 (19.78) |
| Inflammatory bowel disease | 544 (1.97) | 438 (2.74) | 76 (3.50) | 27 (0.30) | --- |
| Bipolar disease | 1260 (4.57) | 1021 (6.39) | 161 (7.42) | 73 (0.82) | --- |
| Schizophrenia | 306 (1.11) | 260 (1.63) | 30 (1.38) | 16 (0.18) | --- |

Table notes: ACO=Accountable Care Organization; FSP=Flexible Services program; --- denotes less than 12 individuals.

^1^Medicaid ACO enrollees were patients aligned to the Mass General Brigham (MGB) ACO.

^2^Screening was completed during primary care health visits or through contact with patients’ primary care clinicians from March 2018 (beginning of annual social needs screening for Medicaid ACO patients) through March 2023.

^3^Eligibility criteria for FSP included: (1) alignment to the MGB Medicaid ACO, (2) food or housing insecurity identified by screening or clinical encounter, and (3) a complex health condition (e.g., uncontrolled diabetes, depression) identified in the electronic medical record that satisfied eligibility criteria for FSP eligibility.

**Supplemental Table 3.** Number, duration, and type of service enrollment for Flexible Services (FSP) enrollees aligned to the Mass General Brigham Accountable Care Organization (ACO), March 2020-March 2023.

|  | **Adults enrolled in FSP^1^**  **mean [SD] or N (%)** | **Children enrolled in FSP^1^**  **mean [SD] or N (%)** |
| --- | --- | --- |
| Mean number of enrollments | 1.40 [0.66] | 1.28 [0.58] |
| Number of enrollments |  |  |
| 1 enrollment | 1648 (66.86) | 500 (77.64) |
| 2 enrollments | 669 (27.14) | 117 (18.17) |
| 3 or more enrollments | 148 (6.00) | 27 (4.19) |
| Mean duration of FSP enrollment (months) | 14.46 [7.60] | 12.34 [5.77] |
| Mean duration of nutrition enrollment (months) | 14.37 [7.28] | 11.66 [5.13] |
| Mean duration of housing enrollment (months) | 10.77 [4.61] | 12.84 [4.40] |
| Enrolled in nutrition services | 2069 (83.94) | 564 (87.58) |
| Medically tailored meals or food box | 637 (25.84) | 226 (35.09) |
| Produce voucher | 1515 (61.46) | 359 (55.75) |
| Enrolled in housing services | 550 (22.31) | 107 (16.61) |
| Enrolled in both nutrition and housing services | 154 (6.25) | 27 (4.19) |

Table notes: ACO=Accountable Care Organization; FSP=Flexible Services program

^1^Eligibility criteria for FSP included: (1) alignment to the MGB Medicaid ACO, (2) food or housing insecurity identified by screening or clinical encounter, and (3) a complex health condition (e.g., uncontrolled diabetes, depression) identified in the electronic medical record that satisfied eligibility criteria for eligibility.
